# The Role of Desmopressin in Kidney Biopsies: A Narrative Review

**DOI:** 10.1177/20543581251366752

**Published:** 2025-11-30

**Authors:** Sheldon W. Tobe, Raea Dobson

**Affiliations:** 1 Sunnybrook Health Sciences Centre, Toronto, ON, Canada

**Keywords:** desmopressin, DDAVP, kidney biopsy, bleeding

## Abstract

Desmopressin is a vasopressin analogue. In addition to its antidiuretic effects, it is also a procoagulant. While it is indicated to reduce bleeding in a variety of situations, it is not currently being utilized broadly for kidney biopsies, a procedure where bleeding is the most common complication.

**Purpose of Review:**

To discuss the evidence surrounding use of desmopressin for kidney biopsy.

**Sources of information:**

We conducted a search of MEDLINE (Ovid), Embase (Ovid), CENTRAL (Wiley), International Pharmaceutical Abstracts (Ovid), and Scopus databases.

**Methods:**

All identified trials were reviewed. Trials since the last systematic review in 2020 were organized in color-coded tables by efficacy, neutral results, or harm.

**Key Findings:**

Studies are generally favorable in terms of efficacy data for reduced bleeding during kidney biopsies, with some safety concerns. More studies are still needed, but we believe desmopressin can be justified to reduce bleeding complications of kidney biopsy in the setting of chronic kidney disease (CKD).

**Limitations:**

Trials involved small sample sizes, single-center data, and were largely observational in nature.

## Abrégé

En plus d’avoir des effets antidiurétiques, la desmopressine, un analogue de la vasopressine, est aussi un agent procoagulant. Actuellement, bien qu’elle soit indiquée dans plusieurs situations pour réduire les saignements, la desmopressine est peu utilisée en contexte de biopsie rénale; une procédure dont l’hémorragie est la plus fréquente complication.

**Objectif de la revue:**

Discuter des données probantes entourant l’utilisation de la desmopressine en contexte de biopsie rénale.

**Sources:**

Nous avons effectué une recherche dans les bases de données MEDLINE (Ovid), Embase (Ovid), CENTRAL (Wiley), International Pharmaceutical Abstracts (OVID) et Scopus.

**Méthodologie:**

Tous les essais répertoriés ont été examinés. Tous les essais menés depuis la dernière revue systématique (2020) ont été organisés dans des tableaux avec codes de couleur pour l’efficacité, les résultats cliniques neutres ou les effets indésirables.

**Principales observations:**

En général, les études montraient des données d’efficacité favorables sur la réduction des saignements pendant les biopsies rénales, bien que présentant quelques préoccupations en matière de sécurité. D’autres études sont nécessaires, mais nous croyons que l’utilisation de la desmopressine pendant la biopsie rénale serait justifiée pour réduire les risques d’hémorragie en contexte d’insuffisance rénale chronique (IRC).

**Limites:**

Les essais examinés portaient sur des échantillons de petite taille, présentaient des données provenant d’un seul centre et étaient en grande partie de nature observationnelle.

## Introduction

Kidney biopsies are the gold-standard diagnostic tool for evaluating the underlying pathology of kidney dysfunction. The most significant complication of inserting a biopsy needle into a highly vascularized organ such as the kidney is bleeding; this ranges from minor bleeding (eg, small kidney hematoma, or self-limited gross hematuria) to major bleeding requiring transfusion and/or an additional interventional procedure to resolve bleeding. Any excess bleeding can cause distress for patients and can lead to increased length of hospital stay, putting further stress on already over-extended health care settings.^1^ Data on the frequency of bleeding after a kidney biopsy comes from a retrospective study from London, Ontario of 617 consecutive adult kidney biopsies (247 native kidneys) with mean creatinine 201 μmol/L.^2^ There was a 12.8% overall rate of bleeding; of these, 10.9% were minor (ie, did not require further intervention), whereas 1.6% were major and required transfusion, surgery, and/or embolization to resolve.^2^ Of note, since routine post-biopsy ultrasound was not performed in this study, the number of asymptomatic hematomas was unknown and bleeding rates were lower than studies including post-biopsy ultrasound to detect “silent” hematomas. Major bleeding complications are less common with normal kidney function, but at lower glomerular filtration rate (GFR), bleeding is more frequent due to multiple factors including platelet dysfunction.^2 [Bibr bibr3-20543581251366752]^

Few medications are efficacious or reduce hemorrhagic risk in chronic kidney disease (CKD). For example, a study of tranexamic acid to reduce the risk of post-biopsy hematomas unexpectedly found that this therapy was associated with a larger risk of hematomas than a placebo.^4^

Desmopressin acetate, otherwise known as 1-deamino, 8-d arginine-vasopressin or DDAVP, is a synthetic vasopressin analog best known for its antidiuretic effects for indications such as diabetes insipidus. However, its lesser-known effects include procoagulant activity. DDAVP is an agonist of the vasopressin-2 (V2) receptor on endothelial cells and platelets via a cAMP-mediated signaling pathway causing exocytosis of Weibel-Palade bodies, the release of von Willebrand factor (vWF) multimers, and factor VIII. Von Willebrand factor binds to circulating factor VIII, protecting it from rapid degradation and preserving its activity in clot formation. Platelet V2 receptor activation also releases vWF and brings glycoprotein 1b/IX (Gp1b/IX) and P-selectin (CD62) to the platelet surface from alpha granules, enhancing platelet adhesion to exposed subendothelial collagen at sites of vascular injury. Gp1b/IX and P-selectin further promote platelet aggregation, strengthening the hemostatic plug. This ultimately results in enhanced platelet adhesion, support of fibrin clot formation, and shortening of the bleeding time in inherited or acquired platelet function disorders.^5^ As such, its procoagulant role has been assessed for reducing bleeding in settings such as trauma patients treated with platelet-inhibiting drugs.^6,7^

A recently published systematic review has provided an update to a previous Cochrane review on the hemostatic efficacy of periprocedural desmopressin.^8^ Lim et al identified 2 studies, one of which was a well-conducted single-center randomized controlled trial (RCT) by Manno et al,^9^ and the other a prospective cohort series by Peters et al.^10^ Manno included patients with serum creatinine <133 μmol/L or estimated GFR (eGFR) >60 mL/min, in contrast to the Peters study with serum creatinine levels >150 μmol/L. Lim concluded that there was insufficient high-quality evidence to support the routine use of DDAVP for percutaneous biopsies. Manno et al^9^ found a significant reduction of 24-hour post-biopsy bleeding, identified by ultrasound. Peters et al^10^ found that desmopressin reduced major and overall biopsy complications but not minor complications (ie, hematuria) including nonbleeding complications such as acute hydronephrosis, infections, and septicemia. Lim also reported that while desmopressin reduces periprocedural bleeding versus placebo, it did not reduce the need for any transfusion, nor did it decrease blood loss or the number of units of red blood cells transfused. For safety, it found a doubling of the risk of hypotension, but no precipitation of hyponatremia or thromboembolic complications.^8^ Lim emphasized that while adverse effects were not increased by DDAVP, the follow-up duration in both studies of 24 hours or less was too short to adequately assess for complications such as thrombosis and hyponatremia, which have a median onset of 3 days post-dose for those with CKD. The half-life of desmopressin is approximately 4 hours in healthy subjects, and 10 hours with eGFR <30 mL/min, which increases the risk for prolonged hyponatremia for those with low kidney function.^11^

Despite the aforementioned procoagulant benefits, desmopressin is not currently widely utilized for hemostasis in kidney biopsy.^9,12^ More attention has been paid recently to desmopressin in recognition to its antitumour effects including reducing tumor angiogenesis^13^ and protecting against tumor cell dissemination.^14^ By reducing plasminogen activator, a key enzyme in the activation of matrix metalloproteases which facilitate cancer invasion and metastasis, desmopressin was found to significantly enhance the efficacy of chemotherapy for prostate cancer.^15^ This has led to greater interest in the role of desmopressin for organ biopsies, including hemostasis in kidney biopsy. We sought to review the evidence for the safety and efficacy of desmopressin for this indication, particularly for patients with CKD, and also to assess whether older retrospective studies were confounded by low eGFR.

## Methods

A comprehensive review of the existing literature on this topic was conducted by the authors. Systematic searches were performed in MEDLINE (Ovid), Embase (Ovid), CENTRAL (Wiley), International Pharmaceutical Abstracts (Ovid), and Scopus databases, from inception to August 2, 2024, using the search terms “desmopressin,” “DDAVP,” “vasopressin,” “antidiuretic hormone,” “deamino-d-arginine vasopressin,” “renal biopsy,” and “kidney biopsy.” References for the located studies were reviewed by the authors for other relevant articles. The purpose of the review methodology was to provide a comprehensive, critical, and objective analysis of current knowledge on the use of desmopressin for kidney biopsies. The choice of a narrative review was driven by the available evidence with only a small number of studies, 3 single-centered RCTs, 3 observational studies, and 6 retrospective analyses, affected by selection bias and methodological variation. The clinical and methodological heterogeneity of study populations, interventions, and outcome definitions made a formal systematic review impractical and potentially misleading. The intent of this review is not to catalog the entirety of the literature, but to critically appraise and synthesize what limited data do exist beyond the last review, to inform clinical thinking and future research priorities, and allow a more flexible and contextualized synthesis of the available studies including identification of knowledge gaps pointing toward further investigation.

Only studies published in English language were included. Study selection was based on relevance to this question with the goal to find systematic reviews, RCTs, prospective cohort studies, retrospective cohort studies, and case series. We excluded case reports and did not extend the review into the gray literature. The 2 authors independently extracted data. Relevant peer-reviewed studies were identified through iterative searching, including backward citation tracking from key articles and related reviews. The STROBE statement for reporting observational studies, and the 2010 CONSORT statement to identify key elements, was used to assist in describing observational studies and RCTs in this narrative review.^16,17^

To provide a visual aid for the reader, a color coding system is used. Studies with favorable efficacy for major or minor bleeding complications with at least a clear trend toward benefit (a large effect size, over 25% if not statistically significant) are shown in green, those with neutral results are shown without color, and those demonstrating harm are shown in red.

## Results

### Randomized Controlled Trials

Two small RCTs that studied the use of desmopressin versus placebo in percutaneous ultrasound-guided kidney biopsies were identified and are summarized in Table 1. They both demonstrate a reduction in bleeding events, largely driven by fewer hematomas found with post-biopsy ultrasound. A third study by Chakrabarti published in draft while this manuscript was in revision is included in Table 1.^18^

**Table 1.** Randomized Controlled Trials of Desmopressin for Percutaneous Ultrasound-Guided Kidney Biopsies.

Author, Country	Manno et al,^9^ Italy^a^	Sattari et al,^19^ Iran	Chakrabarti et al,^18^ India
**Methodology**			
GFR	Creatinine at BL < 1.5 eGFR ≥60	15-90 mL/min by MDRD formula	Creatinine 3.6, eGFR < 60 mL/min
N (% female)	163(45). Adequately powered. Groups appeared well-balanced at baseline.	120 (52). Calculated sample size was 146 subjects, initially attained, but 26 drop-outs occurred post-allocation. Groups were overall balanced at baseline.	152(48). Adequately powered.
Kidney type	Native	Native	Native
Inclusion criteria	16 to 80 years old **Preserved kidney function**: creatinine less than 133 μmol/L and eGFR 60 mL/min or more by MDRD Normal coagulation parameters Blood pressure less than 140/90 mm Hg	18 to 80 years old **Reduced kidney function:** 15 to 90 mL/min by MDRD	Age >18 years BP controlled eGFR < 60 mL/min/1.73 m^2^
Exclusion criteria	Solitary kidney, renal cancer, hydro-, or pyonephrosis, significantly small kidney size on ultrasound, obesity, acute kidney injury. Antiplatelet drugs and heparins were stopped 7 days and 1 to 2 days pre-biopsy, respectively. No mention of oral anticoagulants.	Impaired coagulation (platelets less than 50 × 10^9^ cells/mm^3^, prolonged prothrombin time or INR, blood pressure more than 160/100 mm Hg, solitary kidney, kidney cancer, hydronephrosis, pyelonephritis, small kidneys less than 9 cm on ultrasound, BMI more than 30 kg/m^2^, hemoglobin less than 70g/L, serum sodium less than 135 mmol/L, primary polydipsia, multicystic kidney disease, or transplanted kidney. Antiplatelets and heparin were held 7 days and 2 days pre-procedure, respectively.	Solitary kidney, RCC, hydronephrosis, small kidney (<9 cm), heart disease, thrombotic events, BMI ≥ 40 kg/m^2^, severe nasal congestion, sodium < 125 mEq/L
Desmopressin dose	0.3 μg/kg subcutaneously	3 μg/kg intranasally	3 μg/kg intranasally
eGFR mL/min/Creatinine mmol/L	94.2/88 vs 89.4/87.8	54.1/138 vs 49.4/169	23/3.6 vs 22/3.7
Timing	1 hour pre-procedure	1 hour pre-procedure	1 hour pre-procedure
Placebo	Saline subcutaneously	Saline 0.65% intranasally	Saline intranasally
Needle size	16 gauge automated spring-loaded gun	18 gauge	16-gauge automated spring-loaded gun
Randomization	1:1, allocation concealed, block randomization (blocks of 10) by computer-generated random number list	Allocation concealed via opaque envelopes, block randomization (blocks of 6), computer generated sequences	Block-randomized, computer-generated sequences.
Blinding	All blinded (subjects, investigators, outcome assessors, and nurses who prepared and administering drugs)	All blinded (subjects, investigators, outcome assessors). Investigators opened envelopes only at the time of allocation	All blinded. Vials wrapped in similar paper and marked 1 or 2.
Follow-up period	24 hours	24 hours	24 hours
**Outcomes**			
Primary outcomes reported in the trial	Major: arteriovenous fistula formation, acute renal obstruction, rapid decrease in hemoglobin level requiring blood transfusion, angiography and gel-foam transarterial embolization, sepsis, death Minor: peri-renal hematoma 2 cm × 2 cm or more, gross hematuria that resolved without intervention	Major: arteriovenous fistula, acute renal obstruction, need for blood transfusion, endovascular embolization, sepsis, death Minor: peri-renal hematoma, gross hematuria	Major: blood transfusion, embolization, nephrectomy, death. Minor: flank pain, gross hematuria, perinephric hematoma.
Secondary	Hematoma size, post-biopsy hemoglobin level, coagulation parameters, GFR, blood pressure, length of hospital stay.	Volume of peri-renal hematoma, changes of post-biopsy hemoglobin and hematocrit, plasma sodium, and blood pressure.	Size of hematoma at 6 and 24 hours
**Results**			
Average patient	40-year-old male, 70 kg, blood pressure 127/81 mm Hg, hemoglobin 137 g/L, eGFR 90 mL/min, post-biopsy finding of glomerulonephritis	45-year-old female, BMI 25 kg/m^2^, blood pressure 125/79 mm Hg, hemoglobin 112 g/L, eGFR 52 mL/min, sodium 140 mmol/L, acute on chronic kidney injury.	39-year-old male, BP 131.5/82.5, Hb 9.2, eGFR 22.5 mL/min/1.73 m^2^, sodium 132.5, 4.3 hemodialysis sessions pre-biopsy
Outcomes (as desmopressin vs placebo)	*Post-biopsy bleeding/hematoma formation*: 13.7% vs 30.5% (*P* = .01) ARR: 16.8%, NNT: 6 Hematoma size: median 208 mm^2^ vs 380 mm^2^ (*P* = .006), AR: 172 mm^2^ Hospital stay: 4.9 vs 5.9 days (*P* = .004) Post-biopsy hemoglobin: no change for either group *Safety*: No differences in blood pressure, coagulation parameters, or eGFR. No transfusions, angiography, embolization, or nephrectomy were required. No hyponatremia or thromboembolism. Transient, small increase in heart rate (n = 3).	*Total bleeding events*: 23.3% vs 55% ARR 31.7%, NNT 4 *Post-biopsy peri-renal hematoma*: 11.6% vs 40% ARR: 28.4%, NNT 4 *Hematoma volume*: 2.21 mm^3^ vs 7.72 mm^3^ *Major complications, gross hematuria, post-biopsy hemoglobin and hematocrit, blood pressure, serum sodium*: no statistically significant differences. *Desmopressin-induced adverse effects*: Mild transient headache (3 subjects), self-resolving cutaneous flushing (2 subjects), transient self-resolving tachycardia. No cases of hyponatremia, thrombotic events, or volume overload.	*Overall bleeding*: 36% vs 47% ARR 11%, NNT 9 *Post-biopsy peri-renal hematoma at 6 hours*: 35% vs 46% *ARR 11%, NNT 9* *At 24 hours* 19% vs 36% (*P* = .02) *Hematoma volume at 6 hours*: 1.5 mL vs 2.7 mL *Hematoma volume at 24 hours*: 1.2 vs 2.0 Major complications: blood transfusion 8.1% vs 6.4%, embolization 2.7% vs 1.3%, *Desmopressin-induced adverse effects*: Sodium 130 vs 133 mmol/L Side effect profile, otherwise reported as similar
Limitations	Small sample size, single-center study, subjects at relatively low risk of developing complications, underpowered to detect adverse effects of desmopressin.	Did not meet desired sample size (though was still able to detect meaningful differences). eGFR was high on average for a “reduced kidney function” study.	Single-center study.

*Note.* MDRD = Modification of Diet in Renal Disease equation; eGFR = estimated glomerular filtration rate; AR = absolute reduction; ARR = absolute risk reduction; NNT = number needed to treat; BMI = body mass index; BL = baseline; BP = blood pressure; INR = International normalized ratio; RCC = renal cell carcinoma.

a Included in Lim et al.^8^

Sattari et al^19^ studied people with planned native kidney biopsies, randomizing 120 participants across 2 sites, to intranasal desmopressin or placebo. The mean eGFR was 52 mL/min, which was considerably lower than the 90 mL/min of Manno et al,^9^ and was associated with almost double the overall bleeding events (Table 1). Sattari and Manno found a greater than 50% reduction of total bleeding events with a number needed to treat (NNT) of only 4 and 6 for desmopressin, respectively. However, most of the bleeding events were minor, including peri-kidney hematomas which were reduced from 40% to 11.6% and 30.5% to 13.7%, respectively, by Satari and Manno. There were too few major complications in both studies to find significant differences. From a safety perspective, both the Sattari and Manno studies found no signal for hyponatremia or thrombosis. Chakrabarti et al^18^ studied native kidney biopsies with lower GFR, mean 22.5 mL/min, finding nonsignificantly lower overall bleeding 47% versus 36%, but did find lower sodium levels (130 vs 133 mmol/L) with desmopressin.^18^

### Observational Studies—Prospective or Mixed Prospective/Retrospective

Three observational studies compared bleeding with or without desmopressin based on local institutional practice for kidney biopsies (see Table 2). Two studies demonstrated fewer overall bleeding events, with fewer major bleeding episodes in one, and fewer post-biopsy hematomas in another. There were too few bleeding events to allow a comparison in the third study.

**Table 2.** Observational Studies (Mixed Prospective-Retrospective) of Desmopressin for Percutaneous Ultrasound-Guided Kidney Biopsies.

Author, Country	Peters et al,^10^ Sweden	Peters et al,^20^ Sweden	Rao and Chandra,^21^ India
**Methodology**			
N (% female)	576 biopsies in 527 patients (29%) Desmopressin: 204 No desmopressin: 372	282 patients Desmopressin: 39 No desmopressin: 243	194 subjects (m, f—not stated). Desmopressin: 89 (prospective) No desmopressin: 105 (retrospective)
% prospective	87	98	46
Kidney type	Native	Transplant	Native
Inclusion criteria	Reduced kidney function: SCr > 150 μmol/L (range 151-1948)	Reduced kidney function: SCr > 150 μmol/L	Reduced kidney function: SCr > 132.6 μmol/L and/or eGFR less than 60 mL/min, not receiving dialysis.
Exclusion criteria	None stated	None stated	Known cardiovascular disease, previous thrombotic events, serum sodium less than 125 mmol/L, transplant biopsies. Antiplatelets and anticoagulants held for at least 2 weeks pre-procedure.
Average patient	61-year-old male, BP 135/78, eGFR 22 mL/min, BMI 27	53-year-old male, eGFR 28 mL/min	38 years old, BP 128/76 mm Hg, hemoglobin 112 g/L, eGFR 35 mL/min
Desmopressin dose	0.3 μg/kg subcutaneously	0.3 μg/kg subcutaneously	150 μg intranasally
eGFR mL/min/Creatinine mmol/L	22.3/338 vs 22.4/316		36.58/206 vs 34.92/216
Timing	Pre-procedure (not specified)	Within 1 hour of procedure	~1 hour pre-procedure
Comparator	No desmopressin	No desmopressin	No desmopressin. All subjects were restricted to less than 1.5L of fluid on the day of biopsy.
Needle size	16 to 18 gauge	16 or 18 gauge	16 or 18 gauge (~82% 16 gauge)
**Outcomes**			
Primary	*Major biopsy complications*: need for transfusion or need for another acute intervention *Minor complications*: issues that resolved spontaneously without intervention	*Major complications*: required intervention (eg, transfusion, invasive procedure). *Minor complications*: resolved spontaneously (eg, gross hematuria)	*Major bleeding*: need for transfusion, angiography, or cystoscopy. *Minor bleeding*: gross self-limiting hematuria, peri-nephric hematomas, hemoglobin decrease of more than 10g/L
Results			
Outcomes (as desmopressin vs placebo)	*Overall complications*: 3.4% vs 8.4%, ARR 5%, NNT 20 Women: 0% vs 12.1% *Major complications* (in multivariate analysis): ○ Overall: 2.9% vs 6.5%, ARR 3.6%, NNT 28 ○ Women: 0% vs 8.6% *Minor complications*: no difference No adverse effects	*Overall complication*: 1 vs 17 subjects, not statistically significant *Major bleeding*: 0% vs 3.3%, not statistically significant *Minor complications*: not discussed No desmopressin-induced side effects were detected.	*Overall bleeding*: 15.6% vs 31.4%, ARR 15.8%, NNT 7 *Major bleeding*: not statistically significant, trend toward benefit *Transfusion*: 0% vs 3.8% *Angiography*: 0% vs 0.9% *Perinephric hematomas*: 7.8% vs 18%, ARR 10.2%, NNT 10 Gross hematuria: 7.8% vs 13.4%, ARR 5.6%, NNT 18 Females were found to have higher bleeding risk (OR 2.45). *Serum sodium*: fell in 94% of patients given desmopressin, mean decrease ~4 mmol/L No statistical differences in major bleeding, post-biopsy hemoglobin, or length of stay.
Limitations	Comparator was complication rates at other centers, higher number of passes during procedure for the “no desmopressin” group that could have led to a higher complication rate, did not check serum sodium, biopsies performed by both Nephrologists and Radiologists, no mention of how antiplatelets or anticoagulants were handled.	Mix of nephrologists and radiologists performed procedures, the number of patients with complications was too low to show significant differences despite clear trend toward benefit for major complications, desmopressin was given only in one center then compared to other centers that did not use it, did not check electrolytes to assess side effects.	Single-center, less prospective data than the other studies mentioned here.

*Note.* SCr = serum creatinine, OR = odds ratio; BMI = body mass index; BP = blood pressure; eGFR = estimated glomerular filtration rate.

Peters et al^10^ reported on 576 native kidney biopsies in those with a serum creatinine of > 150 μmol/L; in this study of 3 centers, 1 used desmopressin for all patients pre-biopsy and the other 2 did not. There were only 7 overall complications in the desmopressin group (6 major and 1 minor) while those without desmopressin had 31 (24 major and 7 minor) with a statistically significant improvement in women, but not in men. Post-biopsy hematomas were not routinely assessed.^10^

Peters et al^20^ also published 515 consecutive transplanted kidney biopsies, 75% of which had a serum creatinine of >150 μmol/L (n = 282), where subcutaneous desmopressin was compared to no desmopressin use. None of the subjects given desmopressin had a major complication, compared to 3.3% of those without desmopressin. In terms of minor complications, 1 occurred in the desmopressin group, whereas 9 occurred in the group not given desmopressin. No desmopressin-related side effects were noted.^20^

Following the publication of Peters et al, Rao and Chandra^21^ introduced intranasal desmopressin to their institution. Collecting prospective data on 89 native kidney biopsies for more than 1 year, Rao was able to compare results with 105 people who received biopsies without desmopressin in the preceding year. Overall bleeding was 15.6% with desmopressin versus 31.4% without, mostly driven by perinephric hematomas, which fell from 18% to 7.8% and gross hematuria which fell from 13.4% to 7.8%. No blood transfusion was required after desmopressin, while 3.8% had required a transfusion previously. While there was no statistically significant reduction in major bleeding, there was a trend toward benefit. This study did find that serum sodium fell in 94% of patients given desmopressin (mean decrease 4 mmol/L), all of which were asymptomatic and resolved with fluid restriction.^21^

### Observational Studies—Retrospective

Prospective analyses up to May 2019 were included in the systematic review by Lim et al,^8^ concluding that there was insufficient high quality evidence to recommend desmopressin for bleeding prophylaxis prior to percutaneous kidney biopsies. Since then, 2 RCTs and 6 additional retrospective studies have been reported. The retrospective studies are summarized in Table 3. Overall, these studies included sicker patients with lower kidney function. The results are mixed but consistently demonstrate higher bleeding complication rates for those with lower kidney function.

**Table 3.** Retrospective Cohort Studies of Desmopressin for Percutaneous Ultrasound-Guided Kidney Biopsies.

Author, Country	Ho et al,^22^ Singapore	Cheong et al,^23^ Spain	Jose et al,^24^ India
N (% female), m/f	195	3018 432/344 vs 1087/1155	432 294/114 vs 82/43
Kidney type	Transplant	Native	Native
Inclusion criteria	Adults	Age ≥ 18	eGFR < 30
Exclusion criteria		Transplant kidney	Native
Average patient			
Desmopressin dose	Desmopressin with lower kidney function 0.3 μg/kg	0.3 mg/kg 1 hour before, IV	Intranasal 160 μg 1 hour before
Timing	1 hour pre-biopsy	1 hour pre-biopsy	1 hour pre-biopsy
eGFR mL/min/Creatinine mmol/L	19/280 vs 30.4/190	45/252 vs 73/109 and after propensity score matching 45.0 vs 53.0 mL/min	10.9/537 (53% on dialysis) vs 11.5/516 (54% on dialysis)
Comparator	No desmopressin	No desmopressin	No desmopressin
Needle size	16g	18g	18g
**Results**			
Age		50.2 vs 43.5	39.2 vs 38.9
Outcomes (as desmopressin vs placebo)	Severe hyponatremia in 7 vs none (but none if 1L or less fluid intake) Bleeding 8.2% vs 8.2% Transfusion 4.1% vs 1% Hematoma 1% vs 6.2%	No difference in outcomes after propensity score matching including kidney artery embolization, blood transfusion, total major bleeding, or perinephric hematoma on imaging. Hyponatremia < 125 mmol/L 6% v 2.7%	All bleeding 12.4% vs 20.8% Major bleeding 4.2% vs 3.2% Transfusion 7.5% vs 12% Hematomas 9.8% vs 15.2% Length of Stay (LOS) 8.9 vs 9.6 days
Limitations	Protected high risk patients. But risk of hyponatremia if too much fluid	Sicker patients received desmopressin	Very sick patients. Less overall bleeding, but no change in major bleeding

**Table:** 

Author, Country	Leclerc et al,^25^ Montreal	Tan et al,^26^ Singapore	Athavale et al,^27^ Chicago
N (% female), Desmo vs none	413/328 (79%)	48 (50%) vs 35 (46%)	269 (125/144)
Kidney type	Native	Native	Native 91% Transplant 9%
Inclusion criteria			
Exclusion criteria			
Average patient			
Desmopressin dose		0.2 μg/kg IV 1 hour before	0.3 μg/kg IV, 30 min prior to biopsy
eGFR mL/min/Creatinine mmol/L	28/194 vs 45/123	17.5/246 vs 24.5/198	39/293 vs 65/157
Timing			
Comparator			
Needle size	16g	16g	18
**Results**			
Age	61	66.6	47
Primary			
Results			Increased odds of post-biopsy bleeding with desmopressin (OR 3.88, 1.95-7.74), with decreased bleeding risk for those with creatinine 158 mmol/L or more and increased for those with creatinine less than 158. Hematoma 15% vs 18%).
Outcomes (as desmopressin vs placebo)	Transfusion 6% vs 2% without No severe hyponatremia Symptomatic Hematomas 4% vs 7%	Major bleeding 14.6% v 11.4% All bleeding 27.1% v 17.1% Severe hyponatremia in 8.3%.	Those in the desmopressin group had lower Hb at baseline (104 vs 115 g/L), lower platelet counts (215 vs 245), and longer bleeding time (7.2 vs 4.8 minutes)
Limitations	Much lower eGFR in Desmopressin treated	More bleeding with D, but lower eGFR	

Ho et al^22^ studied transplanted kidney biopsies in 195 people with serum creatinine >150 μmol/L, with 98 subjects receiving intravenous desmopressin. There were 5 major bleeding complications in the group receiving desmopressin compared with 2 in the group without desmopressin, though this could be due to the desmopressin group having a lower mean GFR versus those in the control group (19 and 30.4 mL/min, respectively). There were also 7 cases of severe hyponatremia (defined as serum sodium <125 mmol/L) with desmopressin, and none without. This risk of hyponatremia was lowered if a patient’s fluid intake was less than 1 L on the day of biopsy.^22^

Cheong et al^23^ studied 3018 native kidney biopsies, with 776 receiving desmopressin before the biopsy. Those receiving desmopressin had lower eGFR (45 vs 73 mL/min) were older (age 50 vs 43 years), had higher blood pressure, and lower hemoglobin levels compared with those not receiving it. In the desmopressin group, hematomas were more frequent (5.4% vs 1.7%), and larger, and there were more blood transfusions (11.0% vs 2.8%), as well as a longer length of stay (10.5 vs 6.4 days). When the data were adjusted for eGFR using propensity matching, these differences were no longer statistically significant, but hyponatremia of <125 mmol/L remained statistically significant (6% vs 2.7%).^23^

Jose et al^24^ reported on 432 patients with low eGFR (<30 mL/min) from India, comparing those who had been treated with intranasal desmopressin to those without. The mean eGFR in both groups was 11 mL/min and more than half were already on dialysis, with the majority receiving desmopressin (307 vs 125). Desmopressin reduced any bleeding complications (12.4% vs 20.8%), hematomas (9.8% vs 15.2%), and blood transfusions (7.5% vs 12%).^24^

Tan et al^26^ reported a cohort of native kidney biopsies from 2011 to March 2015 including 83 patients 60 years of age or older with creatinine >150 μmol/L. Those receiving desmopressin (n = 48) had lower eGFR (17.5 vs 24.5 mL/min) and had numerically but not statistically significantly more total bleeding (27.1 vs 17.1%) and major bleeding (14.6 vs 11.4%).^26^

Leclerc et al^25^ studied 413 native kidney biopsies from Montreal, with 79% receiving desmopressin and also having lower eGFR levels compared with those not given desmopressin (28 vs 45 mL/min). Despite the higher bleeding risk, the group receiving desmopressin had a similar likelihood of symptomatic hematomas, and a lower likelihood of urgent radiologic studies.^25^

Athavale et al^27^ reported on 2 groups with widely separated eGFR levels, 39 versus 65 mL/min, finding increased bleeding risk in the lower eGFR group even with desmopressin. See Table 4 for details on GFR differences per trial.

**Table 4.** Summary of eGFR (mL/min) in Retrospective Studies.

Study	Desmopressin use	No desmopressin use	Difference
Ho et al^22^	19	30.4	11.4
Cheong et al^23^	45	73.6	28.6
Jose et al^24^	10.9	11.6	0.7
Leclerc et al^25^	28	45	17
Tan et al^26^	17.5	24.5	7
Lim et al^28^	16.9	27.3	10.4
Simunnov et al^29^	Less than 45	More than 51	Minimum 6
Athavale et al^27^	39	65	26

## Discussion

Vasopressin, otherwise known as antidiuretic hormone, has a plethora of biological effects due to its agonist activity at vasopressin receptors (V1, V2, and V3), resulting in a protective response to acute physiological stress.^30^ Desmopressin acetate (ie, 1-deamino, 8-d arginine-vasopressin or DDAVP) is a synthetic analogue of vasopressin with selective agonist activity at V2 receptors to induce vascular endothelial cells to rapidly increase their production and release of components of the clotting cascade. These factors include factor VIII and the highly multimerized forms of vWF, which is a crucial component of hemostasis via its stabilization of factor VIII to extend its lifespan in addition to enhancing platelet adhesion to the subendothelial basement membrane. The levels of these pro-thrombotic factors increase 3- to 6-fold within an hour of desmopressin administration and have peak effect on bleeding at 90 to 120 minutes. Bleeding normalizes 4 to 8 hours after a single dose, making this a potentially ideal agent to improve short-term bleeding time.^31,32^

Due to its procoagulant effects, desmopressin is used therapeutically for prevention and treatment of bleeding in certain conditions. This is supported by a recent systematic review and meta-analysis on the hemostatic efficacy of peri-procedural use of desmopressin for kidney biopsies that found reduced risk of bleeding events, total blood loss, and the number of units of red blood cells transfused when compared to placebo.^8^ Since it improves bleeding outcomes in other situations, desmopressin has the potential to reduce the risk of bleeding related to kidney biopsies. While it is not used broadly for those undergoing this procedure, there are new data available since the last systematic review on this topic.

In the past, assessing the benefit of desmopressin given prior to percutaneous kidney biopsy to prevent bleeding has been largely limited to retrospective cohort studies which were significantly confounded due to the presence of more substantial kidney dysfunction in the desmopressin groups compared to those who did not receive desmopressin (see Table 4). It is clear from these retrospective studies and the 2 RCTs that there are more bleeding events with lower GFR levels; hence, the results of these studies are obfuscated by the different baseline levels of bleeding risk. Coming to a similar conclusion, a retrospective cohort analysis from a tertiary referral center in Australia reported on 285 kidney biopsies (both native and transplant), with 88 receiving desmopressin. The authors note that a higher incidence of bleeding associated with desmopressin in their study was “likely due to selection bias because desmopressin was administered only to those at higher risk of bleeding.”^33^ The overall conclusion from 2 of the more recent, methodologically robust studies (see Table 1) is that desmopressin reduces bleeding complications, particularly peri-renal hematomas. The recently published manuscript draft of an RCT in patients with very low eGFR, many of whom had received dialysis was powered to find a significant difference, but although there were fewer bleeding complications, there was no statistically significant difference.^18^

In terms of patients who have the highest chance of benefiting from desmopressin use, those with risk factors for post-biopsy bleeding are the logical choice. There are multiple risk factors for bleeding events in this context, listed in Table 5, which include female sex, older age, smaller kidney size, uncontrolled hypertension, prolonged bleeding time, and anti-thrombotic medication use. As previously mentioned, one of the major risk factors for bleeding post-biopsy is low kidney function. The underlying pathology of increased bleeding risk in CKD is multifactorial, and includes dysfunctional platelet-endothelial adhesion as well as reduced platelet aggregation (which is in part due to impairment of the interaction between platelets and vWF).^19^ The RCT by Sattari et al,^19^ and the mixed prospective-retrospective cohort trials by Peters et al,^20^ and Rao and Chandra,^21^ all demonstrated benefit in patients with reduced kidney function for outcomes including overall bleeding.^10^

**Table 5.** Risk Factors for Bleeding in Percutaneous Kidney Biopsy.

Bleeding risk factors
- Known coagulation disorders (eg, hemophilia A) - Prolonged bleeding time (eg, International Normalized Ratio (INR), Partial Thromboplastin Time (PTT), thrombocytopenia) - Female sex - Reduced kidney function (variable definitions) - Use of drugs which increase bleeding risk (eg, antiplatelets, anticoagulants, non-steroidal anti-inflammatory drugs (NSAIDs)) - Hemoglobin low pre-biopsy (eg, less than 120 g/L) - Elderly - Liver disease - Small kidney size - Increased number of needle passes during biopsy - Systolic blood pressure of 130 mm Hg or more - Age greater than 40

Although it appears that desmopressin is beneficial in this setting, it is important to note that it does not completely mitigate bleeding risks from kidney biopsies. All efforts should be made to reduce bleeding risk and negative impacts of bleeding, such as correction of anemia with iron and/or erythropoiesis stimulating agents as appropriate. Also, medications where bleeding is a known side effect should be withheld for appropriate lengths of time pre-procedure (eg, acetylsalicylic acid (ASA), anticoagulants, nonsteroidal anti-inflammatory drugs).^34,35^ See the summary in Table 5 for further details.

One of the most common adverse effects of concern for desmopressin is hyponatremia, which arises from desmopressin-induced free water retention by the kidneys. Lim et al performed post-procedure electrolytes in 423 kidney biopsies, with any level of hyponatremia noted in 39.4% up to 7 days after desmopressin, associated with lower GFR, lower pre-biopsy sodium levels, and a larger desmopressin dosage. Ho noted that fluid intake of less than 1 L on the day of procedure was associated with no cases of severe hyponatremia.^22^ In the RCTs, follow-up was only 24 hours and hyponatremia may have been missed by Manno and Satari, particularly in the patients with lower eGFR. Chakrabarti et al did measure sodium levels at 24 hours finding lower levels with desmopressin, but the desmopressin and control groups had low sodium levels at baseline (132 vs 133 mmol/L).

It seems reasonable to counsel patients receiving desmopressin to limit their free water intake to 1 L on the day of administration, and possibly longer for patients with lower GFR levels. Electrolytes pre-biopsy will help to identify patients with pre-existing hyponatremia who are at highest risk and should be monitored more closely. Other more rare side effects to consider are hypotension, hypervolemia, and thrombosis (including venous thromboembolism, myocardial infarction, and stroke). See Table 6 for those at highest risk of desmopressin-induced side effects.

**Table 6.** Risk Factors for Desmopressin-Induced Side Effects.

Risk factors for side effects	
**Hyponatremia** *Patients should be cautioned to limit fluid intake*	- Baseline low- or low-normal sodium - History of habitual or psychogenic polydipsia - Use of medications known to lower sodium (eg, diuretics, Selective Serotonin Reuptake Inhibitors (SSRIs)) - Syndrome of inappropriate antidiuretic hormone secrection (SIADH) - Liver or kidney failure - Nausea, vomiting, diarrhea
**Hypotension**	- Volume depletion - Antihypertensive use
**Hypervolemia**	Heart failure, hepatic failure, or kidney failure
**Thrombosis** *includes venous thromboembolism, stroke, and myocardial infarction*	- History of thrombosis (eg, antiphospholipid antibody syndrome) - Known thrombophilia - Vascular headaches - History of recent cardiac events or cerebrovascular events - Hypertension

There are limitations to the available evidence. Desmopressin administration in the studies reviewed was given by various doses and routes, including subcutaneous, intranasal, and intravenous, which prevents a direct comparison of efficacy and safety between studies. Also, kidney function levels varied, and it is clear from the data that the bleeding risk is worse with lower eGFR.

The Kidney Health Australia-Caring for Australasians with Renal Impairment Guidelines for Renal Biopsy from 2019 state “No recommendations or suggestions can be made due to lack of evidence.” However, they do advise that this intervention can be considered for “patients with Stage 3b chronic kidney disease and onwards” with higher bleeding risk as compared to those without CKD. Given the risk of hyponatremia, they state “careful attention to fluid balance should be paid . . . and excessive fluid intake should be discouraged for 6-8h after its administration.” They advise avoidance of use in those with a history of significant cardiovascular disease (eg, vascular stent in situ) due to the potential increased risk of thrombosis.^36^ These recommendations seem reasonable, as they focus on using desmopressin for those at highest risk of bleeding, while also attempting to minimize desmopressin-induced side effects.

## Conclusion

Desmopressin has been used for the prevention of bleeding but is not used routinely for kidney biopsies in most centers. New data with 2 new prospective RCTs, 3 prospective observational studies, and retrospective data demonstrate a signal of both efficacy for all bleeding outcomes, including silent hematomas, and safety with appropriate monitoring. However, desmopressin does not completely mitigate the increased bleeding risk from CKD. The retrospective case series used to assess the efficacy of desmopressin in the past are confounded by unequal levels of kidney dysfunction, with desmopressin given more frequently to patients with lower GFR levels who are at higher risk of bleeding complications. While evidence is limited, it supports the need for further study, and the use of desmopressin for kidney biopsy in higher risk patients to reduce both major and minor bleeding outcomes.
